# Polydimethylsiloxane: a new contrast material for localization of occult breast lesions

**DOI:** 10.2478/v10019-011-0009-4

**Published:** 2011-03-29

**Authors:** Geraldo Sérgio Farinazzo Vitral, Nádia Rezende Barbosa Raposo

**Affiliations:** 1 Research Center for Innovative Health Sciences (NUPICS), Federal University of Juiz de Fora, School of Pharmacy, Juiz de Fora, Brasil; 2 Neuroscience Laboratory (LIM 27), Department and Institute of Psychiatry at the University of São Paulo, School of Medicine, São Paulo, Brasil

**Keywords:** breast cancer, surgery, radiological contrast, ROLL technique

## Abstract

**Background:**

The radioguided localization of occult breast lesions (ROLL) technique often utilizes iodinated radiographic contrast to assure that the local injection of ^99m^Tc-MAA corresponds to the location of the lesion under investigation. However, for this application, this contrast has several shortcomings. The objective of this study was to evaluate the safety, effectiveness and technical feasibility of the use of polydimethylsiloxane (PDMS) as radiological contrast and tissue marker in ROLL.

**Materials and methods.:**

The safety assessment was performed by the acute toxicity study in Wistar rats (n = 50). The radiological analysis of breast tissue (n = 32) from patients undergoing reductive mammoplasty was used to verify the effectiveness of PDMS as contrast media. The technical feasibility was evaluated through the scintigraphic and histologic analysis.

**Results:**

We found no toxic effects of PDMS for this use during the observational period. It has been demonstrated in human breast tissue that the average diameter of the tissue marked by PDMS was lower than when marked by the contrast medium (p <0.001). PDMS did not interfere with the scintigraphic uptake (p = 0.528) and there was no injury in histological processing of samples.

**Conclusions:**

This study demonstrated not only the superiority of PDMS as radiological contrast in relation to the iodinated contrast, but also the technical feasibility for the same applicability in the ROLL.

## Introduction

Developed in the late 90’s, the Radioguided Occult Lesion Localization (ROLL) technique came to fill a gap in the preoperative marking of nonpalpable breast lesions. Since its conception at the European Institute of Oncology, the verification of the correct positioning of the technetium pertechnetate associated with human albumin (^99m^Tc-MAA) in breast tissue has been identified as a limiting factor of this technique. To correct this problem, it was recommended that a small volume (usually the same as ^99m^Tc-MAA - 0.2 ml) of radiographic iodinated contrast media be injected with the needle positioned in stereotactic breast tissue together or shortly after ^99m^Tc-MAA. The mammography control being performed immediately after the injection (about five minutes at most), confirms whether or not the marked area matches the suspected area.^1^–^3^

Some operational deficiencies may be identified during the performance of the radioguided procedure that uses iodinated contrast media. Often, there is a radiological image without any precise borders and tissue absorption spreading in a short period of time.^4^ The risks of adverse reactions in patients are also extremely important, such as skin and subcutaneous tissue necrosis and, especially, allergic and anaphylactic reactions with a risk of death. So, there are some situations of failure with a technique considered the gold standard, with variable levels of efficacy (69–95%) in the complete removal of the impalpable lesions.^5^

To correct this ROLL specific stage, the use of other substances that would both provide an appropriate level of radiological contrast (radiopacity) and act as a tissue marker are required. A product that does not migrate after being positioned in the breast and that remains in the same position for a long period of time is understood as a tissue marker, in order to identify or provide the location coordinates of the area to be studied.

There is evidence that shows features of the radiological opacity of polydimethylsiloxane (PDMS) and its ease of identification by different imaging methods such as X-ray and ultrasound^6^, which enables its use as a substitute for the iodinated contrast in the ROLL.

Other studies, primarily in the dermatology area, defined criteria for injection volumes, directions of use, viscosities and other guiding parameters for techniques by which the complications of intra-tissue injection of PDMS occur quite rarely.^7^,^8^

In this paper, the safety, efficacy and feasibility of the use of high viscosity PDMS in the radioguided location of occult breast lesions (ROLL), replacing the iodinate contrast media, were assessed for the first time.

## Material and methods

### Animals

This animal study was approval by UFJF Animals Ethical Committee (protocol nº 006/2007) and was conducted according to the EU Directive and Colégio Brasileiro de Experimentação Animal (COBEA).

The experimental animals used in this study were adult female *Wistar* rats (*Rattus norvegicus,* 140–180 g, 70 days old) maintained under a 12h/12h light/dark cycle at an ambient temperature of 24 ± 2 °C with a free access to standard commercial food and tap water *ad libitum*. After completing one week of acclimation, the rats were divided into five groups of ten animals and used in the experiments described below.

### Acute toxicity

A synthetic polymer (polydimethylsiloxane, 12.500 centistokes of viscosity, medical grade and sterile) (*Saldanha Rodrigues Ltda,* Brazil) in the volume of 0.2 ml was introduced subcutaneously (sc) in the back of healthy *Wistar* rats anesthetized by inhalation of diethyl ether (single dose). After a period of 1, 7 and 14 days treatment they all were sacrificed to collect samples of blood and tissue. Untreated animals (absolute control group) were sacrificed on the same day at the beginning of the experiments and had neither prior management of the animal nor administration of polymer. Those subjected to the puncture in the same area of application of the polymer, but without the injection of the polymer (relative control group), were sacrificed in the same way, 24 h after the puncture.

Rats were observed thoroughly during the first 24 h for the onset of any immediate toxic signs and daily during the 13 day observation period to record any delayed acute effects. The animals were macroscopically examined, aiming at identifying eventual morphological alterations. Some vital organs (heart, liver and kidney) were forwarded for the histological study. At the end of each experiment, all animals were sacrificed by means of inhalation of diethyl ether according to the Ethical Principles of Animal Experimentation (COBEA) and blood samples were collected (fasting for 12 h before sacrifice).

### Body weight

The weight of each rat was recorded on day zero (0) and on the day of the sacrifice of the animals, in their respective groups. In the group “treated 7 days”, the animals were also weighed on day 3. In the group “treated 14 days”, weighing was also conducted on days 3, 7 and 10. The average weight in each group was calculated.

### Haematological profile

Blood samples were collected under 10% EDTA/saline pH 7.2 and examined for hemoglobin concentration, packed cell volume, total erythrocyte count, and total and differential leukocytes counts.

### Biochemical analysis of serum

Blood samples were collected and centrifuged (3000 rpm, 15 minutes, room temperature) and clean sera was separated and collected for the following investigations: serum glucose (glucose-oxidase/peroxidase); urea (UV kinetic); creatinine (Jaffe’s kinetic reaction); uric acid (end-point colorimetric enzymatic); albumin (**c**olorimetric); aspartate aminotransferase **(**AST) and alanine aminotransferase **(**ALT) (optimized ultraviolet kinetic); alkaline phosphatase (colorimetric enzymatic); total cholesterol and triglyceride (colorimetric enzymatic).

### Histopathological studies

Slices of heart, liver and kidney were fixed in 10% buffered (pH = 7.0) formaldehyde, embedded in paraffin wax, sectioned at 5 μm and stained with haematoxylin and eosin (HE). A detailed microscopic examination was carried out in those organs of both control and treatment groups. These analyses were performed at the Laboratório de Anatomia Patológica do Hospital Universitário from the Universidade Federal de Juiz de Fora.

### Human breast tissue

In this study, 32 samples of human potentially healthy breast tissue (n = 16/group) from patients undergoing reduction mammoplasty performed at the Hospital Universitário (HU) from UFJF were used. All patients gave a written informed consent to participate in this study, which was approved by the Human Ethical Committee of the Universidade Federal de Juiz de Fora (protocol nº 0043.0.180.000-07). Samples were sectioned at 3 cm of diameter, simulating an average size of the surgical specimens obtained with the ROLL, and they were cataloged. A small volume (0.1 ml) of high-viscosity polydimethylsiloxane (12,500 cs) was tested as radiological contrast and tissue marker for ROLL.

### Efficacy and technical feasibility of PDMS in human breast tissue

#### Radiological and scintigraphic analysis

Samples (n=32) were divided into 2 groups: control (iodinated contrast) and experimental (PDMS contrast). In the radiology department, the parts were radiographed (Contour Plus, BENNETT-Hologic USA); 0.1 ml of ^99m^Tc-MAA (IPEN – CNEN - Brasil) was injected, which corresponds to the doses of 5 μCi and 0.1 ml of test substances: Omnipaque 300^®^ (Ioexol, Farmasa, Amersham Health Limited*,* U.K.) or PDMS (Saldana Rodriguez Ltda, Brazil), through a needle positioned in the center of the tissue part (same point of ^99m^Tc- MAA injection). In the PDMS group, 0.01 ml of India ink was also injected right after the polymer, so that it was possible to identify the site of injection in the histological processing. Another mammography was performed after one minute of injection, with the same degree of magnification of images.

The specimens were submitted to radioactive counting (scintigraphic) in a gamma camera (ELSCINT model Apex SP6, Israel) for 30 seconds, using a high-resolution collimator at 17 cm distance between the collimator and the parts. The reading area (ROIs) was of 570 pixels. After the scintigraphic phase the pieces were immediately immersed in 10% buffered (pH = 7.0) formaldehyde for 24 hours, for subsequent histological processing.

A comparative evaluation of the contrast quality between the groups (iodinated and polymer contrast) was performed by measuring the largest diameter of contrast area produced in their mammograms, with the aid of a digital caliper. By the quantitative comparison of scintigraphic uptake levels between the groups, the possibility of interference with the nuclear medicine techniques employed in the ROLL was verified.

#### Histopathological studies

The appropriate identification of the tissue and cellular structures adjacent to the polymer (region marked with India ink) was considered indicative of deleterious non-interference in the techniques of preparation and histological analysis.

### Statistical analysis

The statistical analysis was performed using Statistical Package for the Social Sciences (SPSS) 13.0 for Windows. All values are presented as mean ± standard deviation (SD). For the comparisons of continuous variables among the groups a one-way analysis of variance (ANOVA) was employed with individual differences assessed using Bonferroni correction for the multiple comparisons if the ANOVA was statistically significant. The independent *t* test was also used. Two-tailed *p* values < 0.05 were considered to be statistically significant.

## Results

### Animal study

#### Acute toxicity

Rats administered PDMS did not develop any clinical signs of toxicity either immediately or during the post-treatment period. No mortality occurred in both groups (control and treated animals) either immediately or during the 14-days observation period.

#### Body weight

Body weight, gained during the observation period, was comparable to control groups (p=0.879) (data no shown).

#### Haematological profile

Data on the various hematological parameters measured in rats PDMS-treated are presented in Table 1.

PDMS caused no significant alterations in HB, RBC and WBC count, except for the percentage of eosinophils (3.00 ± 1.70 % of total leucocytes, p<0.05). With respect to this cell type, statistically significant difference was found between the absolute control group and the “treated 14 days” group (ANOVA, Bonferroni test), which indicates the beginning of tissue reaction in this group exposed to a long period of contact with the polymer.

#### Biochemical analysis

Data on various biochemical parameters measured in the serum of controls and PDMS-treated rats is presented in Table 2.

There were no marked alterations in any of the specific activities of enzymes, AST, ALT and alkaline phosphatase in PDMS-treated rats. A similar trend of results was observed with regard to the levels of serum constituents, glucose, urea, creatinine, uric acid, albumin, total cholesterol and triglyceride during the experimental period.

#### Histopathology

**FIGURE 1:**
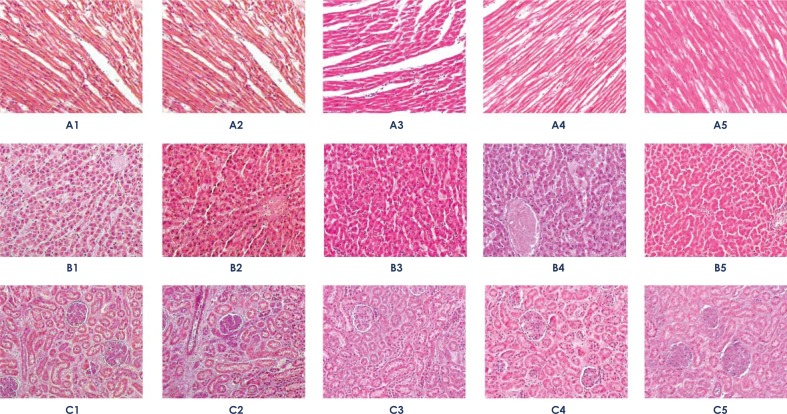
Representative images of heart (A), liver (B) and kidney (C) tissues from animals of all groups stained by hematoxylin-eosin. Sections of these tissues were fixed and stained with hematoxylin-eosin (HE) and examined by light microscopy (magnification: 100×). Numbers represent experimental groups (controls: 1 – absolute and 2 – relative; PDMS-treated animals: 3 - Day 1, 4 - day 7 and, 5 - day 14).

Sections of liver, kidneys and heart tissues of PDMS-animals showed no pathological alterations under light microscopy (Figure 1).

### Efficacy in human breast tissue

#### Radiological and scintigraphic analysis

In human breast tissue, the mean diameter of PDMS-marked tissue was smaller than that obtained with the iodinated contrast group (8.64 ± 2.14 mm *vs.* 17.26 ± 4.91 mm, p<0.001) (Table 3).

**FIGURE 2:**
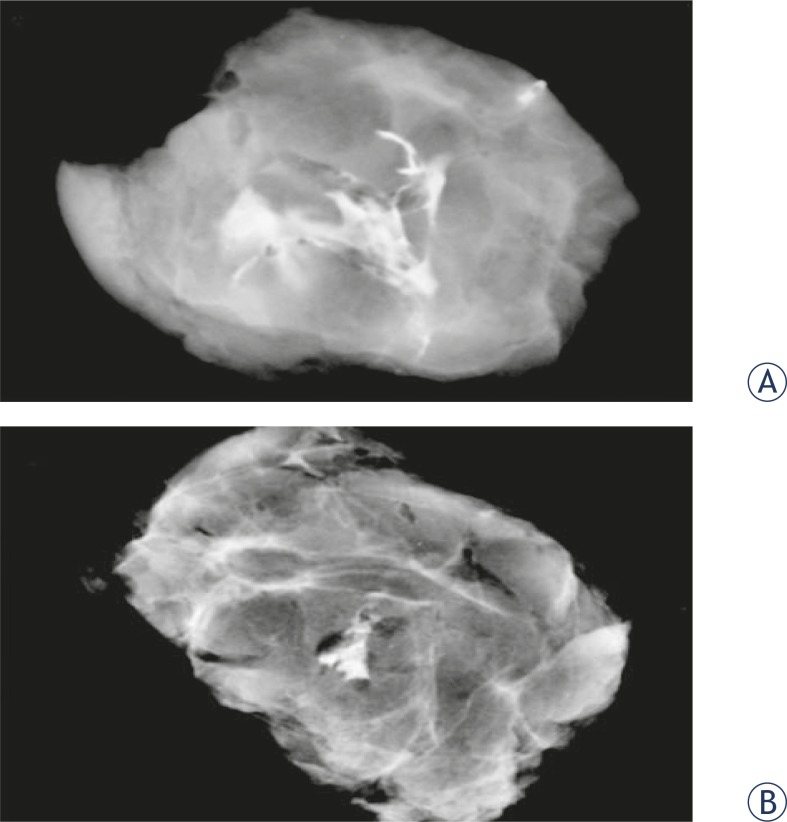
Radiological imaging of a specimen of human breast tissue marked with (A) iodinated contrast and (B) polydimethylsiloxane. Samples were sectioned at 3 cm of diameter and presented at the same degree of magnification.

Figure 2 shows that the PDMS also produced a more regular markup compared to the iodinated contrast.

PDMS did not interfere with scintigraphic uptake (p=0.528) (Table 3).

#### Histopathological analysis

**FIGURE 3:**
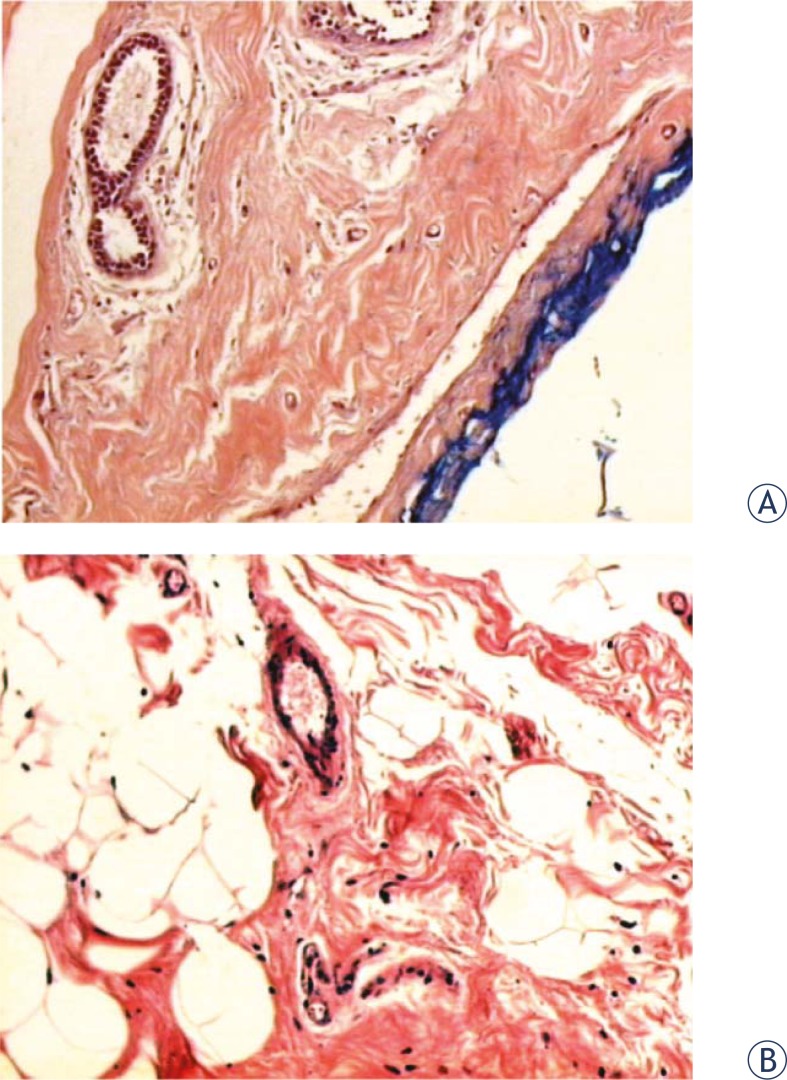
Human breast tissue histology of polydimethylsiloxane (A) and iodinated contrast groups (B). Sections of human breast tissue were fixed and stained with hematoxylin-eosin (HE) and examined by light microscopy (magnification: 100×).

PDMS did not interfere with the histological processing of the breast tissue specimens. Scanty amounts of glandular breast tissue were sometimes observed in both groups, along with abundant adipose tissue, because of the origin of these tissues. In this type of plastic surgery, the emphasis is on the removal of fat, not glandular tissue (Figure 3).

## Discussion

In this study, the possibility of using the synthetic polymer polydimethylsiloxane (PDMS), in lieu of iodinated contrast in ROLL was evaluated. It was assessed aspects of acute toxicity in experimental animals, the quality of radiological contrast and the verification of possible interferences with scintigraphic and histological processing of this technique. The results of this study point to a lack of systemic toxic effects in animals, which confirms other studies about the safety of using this substance in humans.^9^,^10^

Despite the knowledge of the safety in using PDMS in medical procedures, this research is necessary because it is a new use of this product. It is important to highlight that in this new proposed usage, the injected polymer is completely extracted from the body in a short time interval (3–12 h) by the surgery. This extraction of the tissue marker is demonstrated by the use of the gamma portable radiation detector (gamma-probe), intraoperatively. This is because the polymer is injected by the same needle and in the same tissue position, immediately after the injection of ^99^mTc-MAA. This feature further contributes to increase the safety profile of PDMS in this application because, as previous studies have shown^11^–^13^, the possibility of local reactions such as “foreign body” or granulomatous reactions using the PDMS free in the tissue tends to manifest itself in about a week after its administration.

Another aspect to be analyzed with the results of this research refers to the fact that the volume injected into experimental animals was the same as proposed for the use in humans (0.2 ml), without adjustment for weight. This adjustment was not done because it is already a very small volume, which could derail the trial. If it was adjusted for body weight, which is an average of 150 g, the equivalent volume in a 60 kg person would be 80 ml. That is to say that the volume of PDMS used in animals in this study was 400 times greater than that proposed for humans. It would, therefore, be expected that if there was any toxicity caused by PDMS in use, detectable changes would likely occur in the evaluated parameters.

Weight reduction observed in the trial within the same group, although not statistically significant (p<0.05), is probably due to the fasting imposed to carry out the biochemical analysis. According to the “*Guide to the Care and Use of Experimental Animals*” (1980), the required amount of feed per day for each adult rat, considering the amount ingested and waste, is 25 g. This value is close to that found for the difference between initial and final weight for all groups.

The normal range of biochemical parameters in the serum of PDMS-treated rats clearly suggests that PDMS causes no disruption of normal physiological and biochemical homeostasis. Regarding the percentage of eosinophils, the statistical difference between the absolute control group and those “treated 14 days” could indicate the beginning of a tissue reaction, because of the long period of exposure of the animal tissue to the polymer.

Data shows a superiority of PDMS contrast quality, compared with the iodinated contrast, in this application (Figure 2). Marking with PDMS was in all cases more regular and punctual, with less scattering areas through the tissue, in addition to remaining identifiable to the later macroscopic histopathology.

PDMS is a polymer that is highly soluble in xylene, a substance used in the histological processing diafanization stage. Thus, at this stage of histological preparation, most, if not all, of the PDMS is extracted from the tissue, so that in the histological slide the area previously occupied by the polymer will be an “empty” area. That’s why India ink was used in this experiment, so that the correct evaluation of cellular elements juxtaposed to the polymer could be done, confirming the non-interference with the histological processing.

In ROLL, because it is a diagnostic and sometimes therapeutic procedure^14^, the unquestionable need for the histological evaluation of excised tissue is implied. Thus, there would be no sense in using any product or technical step that would undermine the histological processing of surgical specimens. In the present study, no negative interference in routine histological processing was identified. It is noteworthy that the PDMS is insoluble in water, which could result in a waterproofing effect of the tissue, if it is used in inappropriate viscosity (low viscosity). This could affect the process of fixation on the tissue. Thus, it is crucial that the developed technique and the recommended material are used.

The mammary vasculature system and the glandular ductal system itself raise the question of the possibility of embolization of the substances injected during the ROLL. A few cases have been described in which injection and intraductal dissemination of ^99m^Tc-MAA and iodinated contrast occurred.^15^ Such potential exists because of the low viscosity and high solubility of ^99m^Tc-MAA and the iodinated contrast.

Due to the low caliber of the mammary vasculature^16^ and also to the insolubility and high viscosity of PDMS, the risk of embolization would be extremely rare. Possibly, the high viscosity of the polymer could also be a major factor in the relative strengths of the injection pressure and the resistance of the vascular (arterial and venous) walls, much lower than that of a steel needle, which would prevent embolization.

Despite its high viscosity, the PDMS did not interfere with the scintigraphic uptake in the tissues (Table 3). Because it does not reduce the emission scintigraphic tissue marked, it is expected that the use of PDMS does not interfere with the intraoperative detection by gamma-probe, when used in clinical trials.

In conclusion, the analysis of the data suggests that the high viscosity PDMS represents a viable alternative to iodinated contrast in ROLL. The results did not show any toxic effects due to PDMS in animals. Good quality contrast in human breast tissue was obtained, without negative interference detected in the ROLL scintigraphic step and in histological processing of tissues evaluated. Such findings of safety, effectiveness and technical feasibility are essential to carry out assessments in human breast tissue *in vivo*, the objective of future studies.

## Figures and Tables

**TABLE 1:** Haematological data of the control and experimental groups

**Haematological profile**	**Control groups**		**Experimental groups**		
	
	**absolute**	**relative**	**Day 1**	**Day 7**	**Day 14**
Total leucocytes (10^3^/mm^3^)	9.65 ± 2.79	10.61 ± 2.44	8.92 ± 1.48	10.61 ± 2.65	9.27 ± 1.58
Neutrophils (%)	25.60 ± 9.02	20.70 ± 8.06	19.60 ± 6.33	22.60 ± 5.46	20.00 ± 6.80
Lymphocytes (%)	72.00 ± 8.10	75.70 ± 7.13	77.10 ± 6.31	74.70 ± 5.74	75.20 ± 6.39
Monocytes (%)	1.10 ± 0.88	1.90 ± 1.45	1.80 ± 1.23	1.60 ± 1.35	1.10 ± 0.57
Eosinophils (%)	1.20 ± 1.13	1.80 ± 1.23	1.50 ± 1.35	1.30 ± 1.16	3.00 ± 1.70*^a^*
Platelets (10^3^/mm^3^)	577.20 ± 58.60	612.10 ± 75.74	557.00 ± 93.90	617.80 ± 74.26	529.40 ± 87.40
Red blood cels (10^6^/mm^3^)	6.70 ± 1.32	6.84 ± 0.91	6.44 ± 1.19	7.27 ± 1.14	6.02 ± 1.13
Haemoglobin (g dl^−1^)	13.45 ± 2.40	13.54 ± 1.28	12.78 ± 1.95	14.34 ± 2.22	12.47 ± 1.06
Hematocrit (%)	38.43 ± 6.52	39.04 ± 3.90	36.73 ± 5.32	41.04 ± 6.35	37.85 ± 2.78

All values are expressed as mean ± SD.

^a^ p<0.05 (ANOVA followed by *post hoc* Bonferroni).

**TABLE 2:** Biochemical profile of the control and experimental groups

**Biochemical parameters**	**Control groups**		**Treated groups**		
	
	**absolute**	**relative**	**Day 1**	**Day 7**	**Day 14**
Glucose (mg dl-1)	125.0 ± 11.4	119.0 ± 19.5	122.0 ± 20.9	121.0 ± 18.0	118.0 ± 18.0
Urea (mg dl-1)	29.6 ± 3.5	30.8 ± 4.2	29.2 ± 1.8	30.7 ± 4.1	32.3 ± 3.6
Creatinine (mg dl-1)	0.33 ± 0.06	0.38 ± 0.15	0.38 ± 0.06	0.35 ± 0.13	0.39 ± 0.05
Triglyceride (mg dl-1)	76.0 ± 6.2	74.0 ± 5.8	73.0 ± 5.7	74.0 ± 5.6	74.0 ± 5.9
Total cholesterol (mg dl-1)	60.8 ± 6.3	57.2 ± 8.2	56.9 ± 5.6	57.9 ± 8.1	56.5 ± 6.5
Uric acid (mg dl-1)	0.53 ± 0.10	0.53 ± 0.08	0.49 ± 0.10	0.53 ± 0.10	0.52 ± 0.07
Albumin (g dl-1)	2.8 ± 0.3	2.7 ± 0.4	2.6 ± 0.3	2.9 ± 0.3	2.8 ± 0.3
Alkaline phosphatase (Units)	50.6 ± 8.3	51.9 ± 8.0	52.8 ± 8.6	49.5 ± 7.6	47.4 ± 7.5
ALT (Units)	130 ± 10.3	122 ± 9.6	123 ± 8.7	128 ± 8.9	123 ± 6.1
AST (Units)	74 ± 6.0	73 ± 5.9	76 ± 6.8	73 ± 5.9	75 ± 6.9

Results are expressed as mean ± standard deviation (n=10/group). No differences between groups (ANOVA).

**TABLE 3:** The greater diameter of radiological marking and scintigraphic uptake in human mammary tissue with proposed treatments

**Groups**	**Greater diameter (mm)**	**Scintigraphic uptake**
Iodinated contrast	17.26 ± 4.91	1,842.63 ± 560.70
Polydimethylsiloxane	8.64 ± 2.14*^a^*	1,693.00 ± 750.14*^b^*

Results are expressed as mean ± SD (n=16/group).

^a^ p< 0.001 and

^b^ p = 0.528, *Student t test*.
